# The perspectives of nurse-midwives and doctors on clinical challenges of prolonged labor: A qualitative study from Tanzania

**DOI:** 10.18332/ejm/152747

**Published:** 2022-09-19

**Authors:** Aase I. Høifødt, Johanne M. E. Huurnink, Signe Egenberg, Deodatus A. Massay, Bariki Mchome, Tine S. Eri

**Affiliations:** 1 Department of Nursing and Health Promotion, Faculty of Health Sciences, Oslo Metropolitan University, Oslo, Norway; 2 Department of Obstetrics and Gynecology, Stavanger University Hospital, Stavanger, Norway; 3 Independent Health Worker, Mbulu, Tanzania; 4 Department of Obstetrics and Gynecology, Kilimanjaro Christian Medical University College, Moshi, Tanzania

**Keywords:** teamwork, prolonged labor, low-resource setting, health care professionals’ perspective, labor care services

## Abstract

**INTRODUCTION:**

Globally, evidence suggests that one-third of nulliparous women experience delay in the first stage of labor with an increased risk of poor maternal and neonatal outcomes. With this study, we explore how clinical challenges related to prolonged labor are perceived by nurse-midwives and doctors in Tanzania.

**METHODS:**

A qualitative study with group interviews of either nurse-midwives (7 interviews) or doctors (2 interviews). A total of 37 respondents, among them 32 registered nurse-midwives and 5 doctors participated, all with experience from labor wards. A qualitative content analysis was performed. The study setting comprised one zonal consultant university hospital and one regional referral hospital in Northern Tanzania.

**RESULTS:**

Clinical challenges were expressed in relation to: 1) various ways of understanding prolonged labor, manifested by variations in expected duration of labor and the usage of different terms to describe prolonged labor; 2) assessing progress in labor, the partograph being described as an important tool but also a template defining a too narrow normal; 3) appropriate intervention at the appropriate time, the respondents reflect on the correct time for artificial rupture of membranes, oxytocin augmentation and cesarean section; 4) monitoring fetal heart rate, distrust in the monitoring equipment with experiences of surprisingly poor neonates; and 5) working as a team, where the perception of urgency varies and distrust is present.

**CONCLUSIONS:**

Nurse-midwives and doctors in Tanzania face major challenges related to diagnosing prolonged labor, monitoring fetal heart rate and providing high quality health services as a team.

## INTRODUCTION

Diagnosing prolonged labor is complicated by uncertainty related to the definition of onset of labor and normal labor duration^1,2^. The WHO partograph’s alert line represents an expected progress of 1cm cervical dilation per hour and contains a 4-hour action line, which indicates prolonged labor^3,4^. WHO^3^ no longer recommends the use of the alert line to assess satisfactory labor progress, due to its inaccuracy in identifying those at risk of adverse birth outcomes. ‘Slow, yet normal’ has become the new norm, in line with the new WHO Labour Care Guide from 2020^5^.

Up to one-third of nulliparous women may experience delay in the first stage of labor^6^. The most common causes of prolonged labor are either mechanical obstruction or poor or uncoordinated contractions^7^. Prolonged labor increases the risk of infection, postpartum hemorrhage and emergency cesarean section (CS)^8^. Obstructed labor occurs when the fetus cannot descend further in the birth canal, despite good uterine contractions. The most frequent cause of obstructed labor is a mismatch between the fetal head and the mother’s pelvic brim, known as cephalopelvic disproportion (CPD)^9^. In Tanzania, obstructed labor is reported as one of the leading indications for emergency CS and a main cause of maternal and perinatal mortality^10-13^. The 42-day maternal mortality rate in Kenya and Zambia in 2010–2013 was 435/100000 deliveries among women experiencing obstructed labor, prolonged labor or failure to progress compared to 91 in the group experiencing normal progress in labor^14^.

Few studies have assessed the clinical challenges of prolonged labor from a healthcare professional perspective. Several studies address clinical challenges, but mainly those related to management of normal labor and teamwork within the labor ward^13,15,16^. Unlike clinical challenges, structural challenges in labor wards in Tanzania are documented in several studies^13,17-19^. Lack of staff, resources, and equipment, and too few facilities lead to difficulties and delays in providing adequate intrapartum care^20-22^.

The present study is a part of the larger project: ‘Enhancing patient safety in high- and low-resource settings; how to improve the process of decision-making in case of prolonged labor’ (EPSHILS). To strengthen the contextual knowledge prior to complex intervention studies^23^, our objective was to explore how clinical challenges related to prolonged labor are perceived by nurse-midwives and doctors in Tanzania.

## METHODS

### Study design, recruitment and collection of data

The EPSHILS project group used a qualitative design employing semi-structured group interviews. The interview guide was developed by the EPSHILS project group, including contributors from both Tanzania and Norway.

Thirty-two nurse-midwives and five doctors were recruited through purposive sampling by the head nurse-midwives of the obstetric and labor wards at each hospital. The inclusion criteria were: 1) registered nurse-midwife or doctor with experience from the labor ward, 2) currently working within the department of obstetrics and gynecology, and 3) willing to participate in the study. The nurse-midwives’ working experience ranged from recently educated to thirty years, two nurse-midwives had a Master’s degree in midwifery. The doctors had 3–8 years of working experience and represented resident doctors, registrars, and obstetricians and one of them was specialized in Obstetrics and Gynecology. The interview groups consisted of either nurse-midwives (seven groups) or doctors (two groups). In general, the informants had worked mainly in the institution where they were currently employed at the time of the interviews. We did not collect information regarding the informants’ age. In the nurse-midwives’ groups there were 4–5 respondents, the doctors were in groups of 2– 3.

Nine group interviews were conducted between August and December 2018, moderated by the local research assistant of the EPSHILS project, DAM, who is a male clinical officer known to the staff members from a previous research project. Five interviews were conducted at the Regional Referral Hospital (RRH) and four at the Zonal Consultant University Hospital (ZCUH). Each interview was recorded and lasted between 60 and 105 minutes.

### Preparing the material

The material was transcribed and translated from Swahili to English. The transcribed material was anonymized before it was passed on to the authors, AIH and JMEH, who kept a dialogue with the moderator of the interviews, clarifying uncertainties in the material.

### Data analysis

We performed an inductive, qualitative content analysis^24^. AIH and JMEH read through the material several times getting a sense of the whole, and meaning units relevant for our research question were identified^25^. HyperResearch (ResearchWare, version 4.0.3) was used to organize the data. Meaning units were condensed and coded and an iterative process of categorizing was performed, resulting in mutually exclusive, exhaustive, and saturated categories (Supplementary file)^26,27^. All co-authors were given access to the anonymized material and the analysis in order to ensure a mutual understanding of the content.

### Study setting

Tanzania’s healthcare system is decentralized and divided into national, zonal, regional and district levels^28^. The context of this study was one zonal consultant university hospital and one regional referral hospital in Northern Tanzania. Both hospitals receive women referred from district hospitals.

The partograph is started at cervical dilation of 4 cm in the ZCUH and 3 cm in the RRH while the woman is in the antenatal ward. Women are admitted to the labor ward when they have reached cervical dilation of 7 cm. CSs account for 15–20% of the births in the RRH and 35–40% in the ZCUH. Approximately 5% of the laboring women in the RRH are referred to the ZCUH. Both hospitals use fetoscopes (steel/plastic) called Pinard horn, and electronic dopplers for fetal monitoring. There is no access to continuous fetal heart rate monitoring. Oxytocin augmentation is at both facilities administered by infusion, drip rate being manually adjusted due to lack of infusion pumps. The birthing women have no access to adequate pain relief besides opiates. Few women have a companion in the labor ward, although their presence is permitted at both hospitals.

## Results

Clinical challenges were expressed in relation to: 1) various ways of understanding prolonged labor, 2) assessing progress in labor, 3) appropriate intervention at the appropriate time, 4) monitoring fetal heart rate, and 5) working as a team.

### Various ways of understanding prolonged labor

The respondents expressed variations in expected duration of labor. Prolonged labor was understood as going beyond ‘expected hours’. Respondents described the partograph as essential in diagnosing prolonged labor. In the RRH, many respondents operated with a timeframe of twelve hours. One doctor said:

*‘It is labor that goes beyond its usual time which is maximum allowed without having delivered. Let us say twelve hours, beyond that then it is prolonged labor.’* (Doctor, RRH)

At the ZCUH, the expected duration of the active stage for nulliparas ranged from 8 to 24 hours. Respondents in both hospitals differentiated between primiparous and multiparous women for both first and second stage of labor. Nurse-midwives expected the second stage of labor to last no more than twenty minutes to one hour.

Three terms were described explicitly and implicitly – prolonged labor, poor progress in labor and obstructed labor. For medical terminologies, health workers in Tanzania often mix English words with Swahili words. Prolonged labor in Swahili (uchungu uliochukua muda mrefu) may be translated to ‘labor which takes long time’, poor progress in labor (uchungu usioendelea) to ‘labor which does not progress’ and obstructed labor (uchungu kinzani/pingamizi) to ‘labor that is resistant/blocked’. Poor progress was described as an early stage of prolonged labor, but also as an indication for emergency CS, as expressed by this nurse-midwife:

*‘According to my opinion the management of poor progress and prolonged labor are similar, for example in case of prolonged labor I have to establish the causative factor and the management will go from there. But in poor progress, in my management I will directly prepare her for CS ...’* (Nurse-midwife, ZCUH)

Several nurse-midwives associated prolonged labor with severe findings of dry vaginas and restless, febrile mothers. One doctor described a spontaneous vaginal birth as a reason to abandon the diagnosis of prolonged labor:

*‘… I will give her more time, but sometimes we succeed and after some time the mother delivers, you can see it is not prolonged labor.’* (Doctor, RRH)

Descriptions of poor progress, prolonged labor and obstructed labor varied. The three terms were often overlapping and at other times, contradictory. Absence of progress was labelled poor progress by some, and prolonged labor by others. A respondent from the ZCUH said that they often encounter poor progress but stated that prolonged labor usually was referred from other hospitals involving poor outcomes for mother and child. Prolonged labor was also said to cause obstructed labor. Some respondents explicitly addressed confusion among the terms poor progress and prolonged labor. The term ‘early prolonged labor’ (in the latent phase) was also used.

### Assessing progress in labor

The respondents expressed confidence in the partograph – as a guide, indicating time for intervention, avoiding prolonged labor, and helping to prevent poor outcomes. A nurse-midwife said:

*‘Partograph is the first, this is the important tool which I use to know prolonged labor, because it shows from when the mother enters active phase, it shows how contraction goes, it shows fetal heart rate, it shows everything. So, the partograph is a tool we depend on very much and have confidence in.’* (Nurse-midwife, ZCUH)

All groups emphasized the importance of filling the partograph properly. A nurse-midwife in the RRH said that even though they know how to fill in the partograph, often it is not done properly. Some doctors implied that nurse-midwives sometimes filled the partograph in a way that did not indicate prolonged labor. Both doctors and nurse-midwives reflected on how an erroneous first plot in the partograph may wrongly indicate prolonged labor. Nurse-midwives expressed uncertainty in establishing the start of active labor. Referral cases provided additional challenges due to undocumented anamnesis. Despite their confidence in the partograph, doctors at the ZCUH reported that the partograph occasionally forced them to intervene. According to their experience, they sometimes avoided CS by not following the indications of the partograph. Nurse-midwives also experienced the partograph as having a narrow normal range.

Nurse-midwives described vaginal examination and abdominal palpation as a challenge and advocated for training. The respondents used the terms level, descent and station and there was a lack of common understanding of their differences and similarities as this nurse-midwife said:

*‘In measuring spine level, it is a problem … even in interpreting, when you mix interpreting engagement and then comes station, you mix it with level. You have to think twice, which one do you write down? It is zero in spine level but where is it? In plus one, plus two or more? Where is plus two? Where is minus two? It is a problem altogether.’* (Nurse-midwife, RRH)

Nurse-midwives also explained how different finger sizes would lead to differences in cervical dilation measurements. In the ZCUH, nurse-midwives found it challenging how inexperienced doctors sometimes lacked skills in cervical dilation measurement.

Malposition was acknowledged as a cause of prolonged labor. Nurse-midwives in both hospitals regarded it as important, however challenging, to determine the child’s position and presentation. In detecting malposition, respondents also described pain as an indicator. Furthermore, severe pain, lack of pain and unexpected pain made the respondents alert to complications like uterine rupture. Detecting the cause of pain was found to be challenging but essential, as expressed by a doctor:

*‘Why so much pain? … I will go and find out why, why, why.’* (Doctor, RRH)

The respondents described how different expressions of pain influenced how they intervened, sometimes resulting in unnecessary interventions.

### Appropriate intervention at the appropriate time

‘We must take action’ was a frequently used phrase among the nurse-midwives, often synonymous with CS. Respondents said that action must be taken when vaginal birth was not regarded as possible, there was a lack of progress for some time, the child was too big or if the ‘partograph said so’. A nurse-midwife said:

*‘If you use the partograph well, you can know if the things are going well or not, so you can take action. If it is a big baby that definitely cannot come out in a normal way, you must take action as early as possible.’* (Nurse-midwife, RRH)

Furthermore, nurse-midwives described how women asked for CS after being in labor for a long period. However, doctors in both hospitals said that without danger signs, they would not perform a CS solely on request from laboring women. Some doctors said that the fear of performing vacuum extraction was prominent among doctors, leading to an underuse of the procedure.

The respondents mentioned some referral cases where there was said to be prolonged labor and the partograph indicated CS, and still the membranes were not ruptured. When artificial rupture of the membranes (ARM) was performed, the women gave birth. One doctor expressed frustration with guidelines that may indicate early interventions and CS when unnecessary:

*‘… we discuss the difficulty at hand but at the end of the day if I follow my decisions, I find I have helped the mother to have her baby normally; I have not followed guideline.’* (Doctor, ZCUH)

However, for referral cases presenting with dry vagina of high temperature and with the child’s head high, the respondents found that vaginal birth was unlikely to take place.

Regarding oxytocin augmentation, respondents agreed that it should not be administered too early, and that there should be normal fetal heart rate (FHR) in advance. A nurse-midwife at the ZCUH said that the FHR should be monitored ‘most of the time’ when a laboring woman is augmented with oxytocin. Unnecessary induction and inaccurate administration of oxytocin were reported as causes of prolonged labor among the respondents at both hospitals. They expressed a challenge related to the practical administration of oxytocin – whether the valve was sufficiently opened. A doctor explained:

*‘… you will find oxytocin dripping but there is no change in the situation of the mother. It is you who have made the decision of applying oxytocin while she had mild contractions and you have not opened the valve for oxytocin enough for the mother to have strong contractions.’* (Doctor, ZCUH)

The nurse-midwives expressed a need for guidelines regarding the use of oxytocin augmentation as well as when ARM should be performed, although experience had taught them that performing ARM too early would result in prolonged labor, whereas delayed ARM may prevent it. When discussing ARM, nurse-midwives expressed fear of endangering the child by cord prolapse or increased risk of infection, especially transmission of human immunodeficiency virus (HIV).

Discussing the timing for interventions, nurse-midwives addressed the right time for admission to the labor ward. They said that too early admittance may result in misdiagnosed prolonged labor. Too late admittance, as for many referral cases due to repeated delays, made them anxious for both mother and child. A nurse-midwife said:

*‘If prolonged labor happens far away in the rural areas, it takes time to diagnose and to organize. To transport the patient takes time as well. By the time she reaches here, she is tired and also the baby is tired.’* (Nurse-midwife, ZCUH)

### Monitoring fetal heart rate

Respondents from both hospitals conveyed challenges related to finding and interpreting fetal heart rate (FHR). Differentiating FHR from the maternal pulse was found to be difficult, as explained by a nurse-midwife:

*‘It is possible you have listened, but you listened to the maternal heartbeat. You can say there is fetal heartbeat, but if you have not incorporated your colleague or you have not asked the mother if the baby is kicking inside and confirmed it with ultrasound - at the end of the day you come up with such results [stillborn/macerated].’* (Nurse-midwife, RRH)

Several nurse-midwives mentioned incidences where there was said to be FHR, but the child was stillborn. They understood FHR as of either low, normal, or high frequency, and with strong or weak sound. A nurse-midwife described continuous monitoring as listening to the FHR every half hour. Obese or non-cooperative mothers and cases of malpositioned children, represented additional challenges in finding FHR. Some respondents described that they faced no challenges related to the interpretation of FHR, only difficulties in monitoring. Difficulties were closely related to distrust in all available fetal monitoring equipment, as explained by a nurse-midwife:

*‘… there is no answer which I can precisely give if I can rely on either pinard, fetal scope, doppler or even ultrasound - all has its challenges. We have an experience of being told there is fetal heartbeat but on delivery you get a fresh stillbirth.’* (Nurse-midwife, ZCUH)

Nurse-midwives described how dopplers might give faulty readings and mislead them in their work, resulting in children with surprisingly low Apgar score at birth.

### Working as a team

Among the respondents, the perception of urgency varied. Teamwork was perceived as challenging when the doctor gave the laboring women time but forgot to follow up or ‘gave time’ repeatedly. Nurse-midwives in the RRH described how doctors gave more time although they were informed that FHR was negatively affected, as this nurse-midwife explained:

*‘I am the one who knows the patient, I stay with the patient, maybe I have already taken one or two actions. Maybe FHR is 100 or 90 and I can see it is in distress and contractions has slowed down and child cannot come out. I have called the doctor who says we should give her another one hour. Personally, I will tell the doctor … it is not possible to give mother another one hour.’* (Nurse-midwife, RRH)

The doctors said that they only gave time if no danger signs were present for mother or child. They described being available after allowing additional time as a necessity.

Teamwork seemed to be challenged by a mutual distrust between experienced nurse-midwives and inexperienced doctors. In decision-making, doctors were sometimes bypassed if the nurse-midwife did not agree with the decisions made. Nurse-midwives described this bypassing as a way of advocating for the women and children when they felt their conditions were not taken seriously. They argued that increased independence for nurse-midwives, especially when managing uncomplicated labors, would benefit the childbearing women. At the same time, they described fear of being accused of wrongdoing. When fearful of a poor outcome, they put effort into documenting the dialogue with the doctor to minimize the risk of blame.

## DISCUSSION

The purpose of this study was to explore how nurse-midwives and doctors in Tanzania perceived clinical challenges related to prolonged labor. Our findings indicate a mixture of different terms for the phenomenon of prolonged labor. Doubts regarding the accuracy of fetal heart rate findings were evident. Nurse-midwives and doctors had different perceptions of urgency, and dialogue was challenged by distrust.

We found that the terms poor progress, prolonged labor and obstructed labor were discussed as three separate diagnoses, even though the usage was overlapping and at other times, contradictory. An overview of indications for emergency CS in a hospital in Tanzania listed poor progress of labor, big baby, cervical dystocia, prolonged labor and CPD as different indications – poor progress being by far the leading cause for CS^29^. This may indicate that poor progress is used as a broader, less strict diagnosis, encompassing more than what obstructed or prolonged labor does. According to our study, prolonged labor and obstructed labor tend to be used as equivalent terms. One informant said that a vaginal birth would not be labelled prolonged labor, which might indicate an understanding of prolonged labor closely related to mechanical disproportion. The respondents associated prolonged labor with findings of dry vaginas, and restless, shouting and febrile mothers. These findings reflect dehydration and maternal exhaustion, frequently observed in cases of severe prolonged labor or obstructed labor in these settings.

The respondents viewed the duration of normal labor differently. Arriving at a definition of prolonged labor, presupposes an agreement about what constitutes normal labor onset and progress. When this study was conducted, there was no global consensus about the onset and duration of the different stages of labor^2^ or a definition of prolonged labor^30^. According to the respondents, the diagnosis of prolonged labor was given when the ‘expected’ or ‘prescribed’ hours were passed, or the action line of the partograph was crossed.

This study found that the respondents had confidence in the partograph, but at the same time regarded it as presenting a narrow normal. The routine use of the partograph has been promoted by the WHO^3^. However, the validity of the alert and action lines has been called into question during the last decade^31^. The Labour Care Guide^32^ aims to facilitate ‘slow, yet normal’ progression in labor^3^. The respondents’ thoughts were in line with the current waves of research. Similar to Miltenburg et al.^33^, we found that nurse-midwives allegedly chose to chart findings on the partograph in a way that did not indicate prolonged labor, thus giving the laboring women more time to progress.

Crossing of the action line prompted the respondents to ‘take action’, in line with the intention of the partograph. However, ‘taking action’ held ambiguous meaning. At times, the action line alone seemed to be interpreted as an indicator for CS, with the risk of performing CS on a poor indication^10^. Our study found uncertainty around the appropriate timing for other available interventions such as oxytocin augmentation and ARM to facilitate labor progress. In Zanzibar, locally tailored guidelines were implemented, reserving intrapartum oxytocin augmentation until crossing of the action line^34^, leading to significant positive effects on neonatal outcomes.

Our study found a distrust in fetal monitoring equipment, caused by their recurrent experiences of being surprised by poor neonatal outcomes, and similar to the findings of Mdoe et al.^35^. Several of the nurse-midwives explained that interpretation of the FHR was not difficult, which may suggest a simplified understanding of a complex field. Lack of trust in FHR monitoring coexists with a questionable administration of oxytocin. Oxytocin augmentation can induce fetal distress^36^, however, our respondents mainly focused on the risk of uterine rupture. We question if this might imply an underestimation of the risks associated with oxytocin augmentation.

The concept of urgency seemed related to different danger signs: crossing of the action line, exceeding the ‘prescribed hours’, fetal distress or maternal distress. The assessment of maternal wellbeing seemed to be focusing on pain that might imply urgency due to uterine rupture or difficulties like malpositioned child. The respondents often had different perceptions of urgency. According to the nurse-midwives, the doctors would give the laboring women ‘more time’ despite the nurse-midwives’ evaluation of urgency to the situation. The doctors explained that they always evaluated the larger picture before allowing more time for progress of labor. According to the nurse-midwives, they occasionally had to wait a long time for the doctor to arrive, although they had informed about a poor FHR.

Situations where fetal distress was not recognized by nurse-midwives or acknowledged by doctors, may be seen as examples of what is described as ‘too little too late’ in obstetric care^20^. In Tanzania, the decision-to-delivery interval is found to be close to one hour^10,29^. In accordance with other studies from Tanzania^17,33^, the interviewed nurse-midwives were afraid of being blamed in cases of poor outcomes and felt that their competence was underestimated. This may lead to further delay in the diagnosing and treatment because the doctors did not trust the observations of the nurse-midwives^17^. We question if signs of distrust within the team and different perceptions of urgency, as found in our study, might negatively influence the quality of the obstetric care provided by the team as a whole.

### Strengths and limitations

The main strength of this study is that it conveys both nurse-midwives’ and doctors’ perspectives on their management of prolonged labor and what they find clinically challenging. Cross-cultural research carries a risk of missing or distorting information. Attempts were made to limit misinterpretations through recurrent dialogue and close cooperation with the project group of EPSHILS in Tanzania.

## Conclusions

This study provides a broader understanding of the clinical challenges faced by nurse-midwives and doctors at two hospitals in Tanzania, when managing prolonged labor. We identified clinical challenges relating to different ways of defining prolonged labor, assessing progress in labor, the timing and choice of interventions, monitoring FHR and working in a team. The contextual knowledge gained from this study can contribute to locally tailored intervention studies.

## Data Availability

The data supporting this research are available from the authors on reasonable request. An earlier version of the article has been submitted as a preprint at Research Square, at https://doi.org/10.21203/rs.3.rs-74813/v1
