# Supplementary material for: The perspectives of nurse-midwives and doctors on clinical challenges of prolonged labor: A qualitative study from Tanzania
Source: Eur J Midwifery. 2022 Sep 19;6:61. doi: 10.18332/ejm/152747 (PMC9483773; doi:10.18332/ejm/152747)
Supplement: Supplementary file 1 [file EJM-6-61-s1.pdf]

SUPPLEMENTARY FILE

Codes	Subcategories	Category
When mother tell us about a problem, we need proper examination and immediate action<br>If referred mother says she's tired and had long labour, she needs hastly action<br>We take action after checking partograph.<br>No development in 3 PVs need fast action<br>Action must be taken when head is not descending despite enough contractions<br>Some have strong contractions but not dilation - then you have to take action<br>Partograph leads to action and if baby is big you must take early action	Take action	Using appropriate intervention at appropriate time
Early admittance exhausts mother<br>Early admittance ends in PL<br>For referrals there is a long process of diagnosing PL, organizing transport and do the transport.<br>If referred PL case, the baby can be tired on arrival and can have poor outcome.<br>wrongly early admission is bad for the mother	Too early or too late admission	
Delayed ARM prevents PL<br>timing ARM<br>Sometimes membranes can rupture on its own<br>tell us when to rupture membranes<br>ARM is done from 4cm<br>ARM can be enough, some also need oxy<br>ARM is helpful if level is ok<br>We can not do ARM before 6-7cm<br>ARM at 7-8 cm gives quick descent<br>ARM-results are 50/50<br>ARM can detect meconium<br>ARM from 7cm<br>ARM can help to identify position<br>ARM will not make big baby descend<br>ARM at 8-9cm prevents cord<br>Suspect nurses delay ARM because they worry about PL<br>Membranes can be blocking the passage causing PL<br>ARM add to fetal heart rate about baby's condition<br>If we don't do ARM we don't know how baby is doing	When to perform artificial rupture of membranes	
Time of oxytocin administration<br>Dilation in favour of oxytocin<br>timing oxytocin<br>Oxy at 3cm will fail<br>Before no oxy at night, now anytime	When to administer oxytocin	

Before oxy until 6pm, now one nurse is alone in monitoring oxy<br>Oxytocin if foetal heart rate is ok<br>Oxytocin and extraction if no progress<br>What is to be done when mother is stagnant but already on oxy?<br>On oxy you need to hear foetal heart rate most of the time		
Early ARM leads to PL<br>ARM when HIV gives problem to child<br>ARM and oxy at 2-3cm will give delay<br>Alert when silence after oxytocin<br>No ARM in HIV-patients<br>No ARM in HIV-patients<br>ARM can not be done with head high<br>oxy can cause overstressed uterus<br>improper administration of oxy can give PL<br>Early application of oxy causes PL<br>Early ARM can cause baby infection<br>ARM can give cord prolapse<br>Early ARM can risk the baby's life<br>ARM can cause PL<br>unnecessary induction causes PL	Risks associated with artificial rupture of membranes and oxytocin	
Scared of vacuum, not put into good use<br>If you do vacuum extraction you have to be sure the baby will come out	Use of vacuum extraction	
Referred PL needs only ARM to birth, parto says c-section<br>If PL we consider CS 2 hours after starting oxytocin<br>Referral case called PL and with 3cm dilation but no danger signs was sent straight to operation<br>If they stay long they should get CS<br>Mother refused operation and got normal birth<br>If early interventions are done, only CS is left<br>CS depends on cause of PL<br>If no descent on 10cm you might have to do CS<br>If PL I think we might need CS, I call doctor<br>Referral with hot vagina and high level can not get trial of labour<br>Too big baby for the pelvis means CS<br>Those with OPP and transverse will get PL and CS.<br>Partograph says c-section but only need for ARM	Use of caesarean section	
Woman wants oxy to shorten labour<br>Sometimes decision is affected by mothers demands - we have humanity<br>Many ask for operation<br>Listen to those with HIV when they want CS	Mothers want interventions	

Some think crying will give operation<br>If no danger, we don't give CS on request<br>If mother wants CS, but no danger signs, I reassure her she will be fine<br>If mothers wish is not the best for her, we don't approve<br>Some patients say if you cry much you will have operation.<br>Little knowledge of labour pain may lead to women demanding c-section		

© 2022 Høifødt A. I. et al.
